# Vitreoretinal specialists compared with residents on outcomes of primary laser retinopexy in preventing retinal detachment in 958 eyes

**DOI:** 10.1136/bmjophth-2021-000859

**Published:** 2022-02-04

**Authors:** George Moussa, Emma Samia-Aly, Soon Ch'ng, Kim Son Lett, Arijit Mitra, Ajai K Tyagi, Ash Sharma, Walter Andreatta

**Affiliations:** 1 Academic Unit of Ophthalmology, University of Birmingham College of Medical and Dental Sciences, Birmingham, UK; 2 Birmingham and Midland Eye Centre, Birmingham, UK; 3 Kantosspital Winterthur, Brauerstrasse 15, 8400, Winterthur, Switzerland; 4 University of Zurich, Rämistrasse 71, 8006, Zurich, Switzerland

**Keywords:** retina, medical education, vitreous, treatment surgery, treatment lasers

## Abstract

**Objective:**

Retinal tears are the most common vitreoretinal (VR) emergency and retinopexy aims to reduce the risk of rhegmatogenous retinal detachment (RRD). Currently retinal laser is a required competence by the Royal College of Ophthalmologists for residents. We report 6-month detachment rate and repeat retinopexy rate of VR specialists compared with residents.

**Methods and analysis:**

A retrospective, consecutive study of 958 eyes undergoing primary laser retinopexy (slit lamp or indirect laser) from January 2017–2020 was divided into training level by operator: specialty training (ST) 2–3, ST4–5, ST6–7 and VR specialists.

**Results:**

We report an overall 6-month RRD rate in 32/958 (3.3%) (ST2–3: 9/221 (4.1%), ST4–5: 15/373 (4.0%), ST6–7: 2/72 (2.8%) and VR specialists: 6/292 (2.1%)). We additionally report a repeat retinopexy rate of 189/958 (19.7%), (ST2–3: 44/221 (19.9%), ST4–5: 80/373 (21.4%), ST6–7: 16/72 (22.8%) and VR specialists: 49/292 (16.8%)]). Multivariable Cox survival regression analysis showed significant risk factors for developing RRD include male gender (p=0.018), high myopia (≤−6.00 Dioptres, p=0.004), ST2–3 (p=0.022) and ST4–5 (p=0.040) (relative to VR specialists) and by ST6–7, no significance was found (p=0.151). Significantly higher repeat retinopexy rates were associated with horseshoe tears (relative to round holes, p<0.001) and high myopia (p=0.026) with no difference between different training levels.

**Conclusion:**

There was a decreasing trend in RRD rate following primary retinopexy with increase in training. Although junior residents had a higher RRD rate than VR specialists, it was still favourable relative to other large case series. While there was no difference in subsequent laser retinopexy rate between training levels, the retreatment rate was associated with the type of tear and high myopia.

**Key messages**

**What is already known about this subject?**

- Existing large case series demonstrate a rhegmatogenous retinal detachment (RRD) rate following primary retinopexy performed by residents of between 6.9% and 8.8%.

**What are the new findings?**

- Senior residents have a comparable RRD rate following primary retinopexy to vitreoretinal (VR) specialists.
- Junior residents, although having a higher RRD rate then VR specialists, demonstrated an excellent safety profile of primary retinopexy compared with other large case series.

**How might these results change the focus of research or clinical practice?**

- Our findings encourage junior residents to undertake laser retinopexy with adequate supervision.
- Retinopexy outcomes can continue to improve over 6 years of surgical training.

## Introduction

Retinal tears are the most common vitreoretinal (VR) ophthalmic emergency, with a primary aim of retinopexy to reduce the risk of rhegmatogenous retinal detachment (RRD). Laser retinopexy can be carried out in multiple forms: (1) slit lamp retinopexy and (2) indirect retinopexy. Currently retinal laser is a required competence by the Royal College of Ophthalmologists (RCO) for residents, from year 3 of their specialty training (ST) (second year of residency).^1^ Slit lamp laser, being the most common and accessible form of laser, gives increased opportunity in developing competence. It, therefore, becomes a mainstay and integral part of general ophthalmology training, particularly in retinal firms. Moreover, competence of indirect retinopexy usually requires additional training as it is a more challenging technique to master, and it is not separately assessed in the RCO training curriculum.

The National Ophthalmology Database group demonstrated how a reduction in posterior capsule rupture rate following cataract surgery could be achieved with increased level of training.^2^ This emphasises the importance of adequate supervised and unsupervised practice to improve outcomes. In order to demonstrate whether the same principle can be applied when gaining the technical skills required to perform laser retinopexy, we conduct a large case series of primary laser retinopexy to evaluate the 6-month detachment rate following primary retinopexy between VR specialists and residents with a national training number as well as differences in indications for laser (different retinal break morphology) and rate of repeat retinopexy.

## Methods

We present a single centre, retrospective, continuous comparative study of all patients who had primary laser retinopexy from January 2017 to 2020 at Birmingham and Midland Eye Centre, in the UK. All data were extracted from electronic patient records (Medisoft Ophthalmology, Medisoft Limited, Leeds, UK). The research adhered to the tenets of the Declaration of Helsinki and all patient data extracted were anonymised for analysis.

Our cohort of patients was divided into four groups: primary retinopexy performed by ST2–3, ST4–5, ST6–7 and VR specialists. VR specialists were defined as VR fellows and consultants. No VR associate specialists or non-fellows operate in our unit. The most junior residents, on completion of retinopexy, would typically have the application reviewed by a senior resident, otherwise follow-up in the VR clinic would be arranged. If a senior resident/clinician took over to complete the retinopexy, the most senior clinician would be labelled as the operator.

Our primary outcome measure was to compare RRD rate within 6 months following primary retinopexy by training grade. We also assessed the requirement for further retinopexies following primary retinopexy by training grade as secondary outcomes. All patients who had prior VR surgery were excluded. RRD rate was defined as requiring RRD surgery within 6 months of having primary retinopexy in the same eye. Retinopexy of retinal breaks was achieved with laser, via a slit lamp or indirect method and consisted of surrounding retinal breaks with several rows of confluent white laser burns using either a contact lens or a non-contact condensing lens system. Indirect laser was generally used in patient’s intolerant of slit lamp retinopexy (requiring additional local anaesthetic and difficult positioning), small pupils, specific pseudophakic patients (due to the intraocular lens edge obstructing the view of the tear), anterior breaks requiring scleral indentation and retinal tears present with concurrent vitreous haemorrhage where indentation helped provide a view. All patients undergoing primary retinopexy were reviewed within 4 weeks, depending on clinical urgency, in a VR specialist clinic. If patients were subsequently found to have inadequate chorioretinal scar cover, further retinopexy was applied. Patients occasionally presented to our emergency eye clinic with deteriorating symptoms and could be clinically determined to require further retinopexy. All the cases that required retinal detachment surgery following primary retinopexy were performed with transconjunctival 23-gauge pars plana vitrectomy, vitreous-base trim and cryotherapy/laser retinopexy or cryotherapy and/or scleral buckling. As a tertiary referral centre, patients whose postcode was outside, our catchment area was excluded as these patients may have had further retinopexy or surgery at the referring unit. Preoperative data collection included indication for retinopexy: retinal break morphology (horseshoe tears (HST), operculated breaks, round holes), lattice degeneration, RRD (treated with retinopexy alone), other), treatment modality (slit lamp and indirect laser) age, gender and presence of high myopia (≤−6.00 Dioptres). Postoperative data collection included the 6-month RRD rate and repeat retinopexy rate.

### Statistical analysis

Statistical significance was defined as p<0.05. Prior to analysis, normality of continuous variables was assessed using the Shapiro-Wilk test and found not to be normally distributed. Hence, data are primarily reported as medians and IQRs throughout. Kruskal Wallis test was used to compare three or more independent groups, respectively, for continuous variables. A χ^2^ test was used for nominal variables with three or more categories. There were multiple factors that could affect outcome (age, gender, tear morphology (HST compared with round holes), presence of high myopia) and selection bias of treatment modality. Therefore, a multivariable Cox regression survival analysis was performed analysing both repeat retinopexy and RRD rate. Time in days to repeat retinopexy and RRD were used, respectively, with training grade (ST2–3, ST4–5 and ST6–7 compared with VR specialist as reference covariate), gender, age, high myopia and treatment modality (indirect retinopexy compared with reference of slit lamp laser) and indication for treatment (HST vs hole), as covariates.

All statistical analyses were performed using IBM SPSS Statistics for Windows, V.27.0 (IBM, Armonk, New York).

## Results

A total of 958 eyes of 914 patients with primary retinopexy were analysed. Within the resident group, we included outcomes from 54 residents and 18 VR operators over 3 years (some operators are in both groups who performed retinopexy as residents, then in later years, as VR fellows). A summary of demographics and baseline clinical characteristics of primary retinopexy between the training groups are found in table 1. We found a significant difference in the age of patients between different training levels and VR specialists. Pairwise comparisons between groups (Kruskal Wallis test) found a significantly lower patients’ age in the VR specialists’ subgroup compared with each one of the residents’ subgroups, while no significant age difference was detected between the residents’ subgroups. The proportion of men and women which required treatment was significantly different when comparing the VR specialists’ and the residents’ subgroups (table 1, p=0.040). In addition, in the VR specialists’ group, retinopexy was more frequently performed as barrier retinopexy for retinal detachment, in cases of lattice degeneration and retinal holes compared with the residents’ subgroups (table 1). There was also a significant difference in mode of retinopexy between groups (table 1, p<0.001). Residents performed mostly slit lamp laser in 656 (98.5%) compared with VR specialists in 152 (52.1%) of patients (p<0.001). Indirect laser retinopexy was performed in 10 (2.2%) of the residents’ group compared with 140 (47.9%) in the VR specialist group (p<0.001). In addition, VR specialists were more likely to perform bilateral retinopexy than residents (p<0.001).

**Table 1:** Baseline clinical characteristics of primary retinopexy by grade of training

	Total	ST2-ST3	ST4-ST5	ST6-ST7	VR	P value
Total	958	221	373	72	292	–
Age (years, IQR)	59.0 (49.0 to 65.0)	59.0 (52.0 to 65.0)	60.0 (53.0 to 65.0)	61.0 (52.0 to 66.5)	55.5 (33.0 to 65.0)	**<0.001**
Gender (% male)	510 (53.2%)	106 (48.0%)	196 (52.5%)	34 (47.2%)	174 (59.6%)	**0.040**
Laterality (% right)	484 (50.5%)	114 (51.6%)	197 (52.8%)	36 (50.0%)	137 (46.9%)	0.492
High myope (% yes)	33 (3.4%)	3 (1.4%)	16 (4.3%)	1 (1.4%)	13 (4.5%)	0.139
Indication						
*HST*	684 (71.4%)	171 (77.4%)	300 (80.4%)	56 (77.8%)	157 (53.8%)	**<0.001**
*Operculated break*	71 (7.4%)	16 (7.2%)	37 (9.9%)	9 (12.5%)	9 (3.1%)	**0.003**
*Round hole*	85 (8.9%)	19 (8.6%)	26 (7.0%)	4 (5.6%)	36 (12.3%)	0.072
*Retinal detachment*	42 (4.4%)	1 (0.5%)	0 (0.0%)	0 (0.0%)	41 (14.0%)	**<0.001**
*Lattice degeneration*	44 (4.6%)	9 (4.1%)	5 (1.3%)	3 (4.2%)	27 (9.2%)	**<0.001**
*Other*	32 (3.3%)	5 (2.3%)	5 (1.3%)	0 (0.0%)	22 (7.5%)	**<0.001**
Retinopexy type						
*Slit lamp*	808 (84.3%)	219 (99.1%)	370 (99.2%)	67 (93.1%)	152 (52.1%)	**<0.001**
*Indirect*	150 (15.7%)	2 (0.9%)	3 (0.8%)	5 (6.9%)	140 (47.9%)	**<0.001**
Performed bilateral (%)	45 (4.7%)	2 (0.9%)	12 (3.2%)	0 (0.0%)	31 (10.6%)	**<0.001**

Age is reported as median (IQR) and Kruskal Wallis test used to compare continuous variables.

Statistical significance in bold.

Column percentages are reported.

HST, horseshoe tear; VR, vitreoretinal.

A summary of outcomes of primary retinopexy by training level is found in table 2. We report an overall 6-month RRD rate of 32/958 (3.3%) (ST2–3: 9/221 (4.1%), ST4–5: 15/373 (4.0%), ST6–7: 2/72 (2.8%) and VR specialists: 6/292 (2.1%), p=0.481). We additionally show an overall repeat retinopexy rate of 189/958 (19.7%) (ST2–3: 44/221 (19.9%), ST4–5: 80/373 (21.4%), ST6–7: 16/72 (22.8%) and VR specialists: 49/292 (16.8%), p=0.460). The most junior resident group (ST2–3) had the shortest duration from the time retinopexy was performed to the development of a retinal detachment with a median of 4 days (IQR3.0 to 7.0, table 2).

**Table 2:** Outcome of primary retinopexy patients by training level

	Total (958)	ST2-ST3 (221)	ST4-ST5 (373)	ST6-ST7 (72)	VR (292)	P value
6-month detachment (% Yes)	32 (3.3%)	9 (4.1%)	15 (4.0%)	2 (2.8%)	6 (2.1%)	0.481
Further retinopexy (% yes)	189 (19.7%)	44 (19.9%)	80 (21.4%)	16 (22.2%)	49 (16.8%)	0.460
0	769 (80.3%)	177 (80.1%)	293 (78.6%)	56 (77.8%)	243 (83.2%)	0.640
1	156 (16.3%)	39 (17.6%)	64 (17.2%)	16 (22.2%)	37 (12.7%)	
2	25 (2.6%)	4 (1.8%)	12 (3.2%)	0 (0.0%)	9 (3.1%)	
3	6 (0.6%)	1 (0.5%)	3 (0.8%)	0 (0.0%)	2 (0.7%)	
4	2 (0.2%)	0 (0.0%)	1 (0.3%)	0 (0.0%)	1 (0.3%)	
Days to second retinopexy	14.0 (2.0 to 43.0)	9.5 (2.0 to 27.0)	13.0 (2.0 to 36.0)	20.5 (2.0 to 50.5)	23.0 (7.0 to 68.0)	0.083
Days to detachment surgery	15.0 (3.0 to 54.0)	4.0 (3.0 to 7.0)	27.0 (3.0 to 55.0)	45.5 (14.0 to 77.0)	15.5 (4.0 to 55.0)	0.535
Further vitrectomy (% yes)*	29 (3.0%)	9 (4.1%)	13 (3.5%)	2 (2.8%)	5 (1.7%)	0.584
Further buckle (% yes)*	5 (0.5%)	0 (0.0%)	4 (1.1%)	0 (0.0%)	1 (0.3%)	0.383

Continuous variables are reported as median (IQR) and Kruskal Wallis test is used to compare these.

Statistical significance in bold.

Column percentage reported.

*Two patients had combined scleral buckle and vitrectomy.

**Figure 1:**
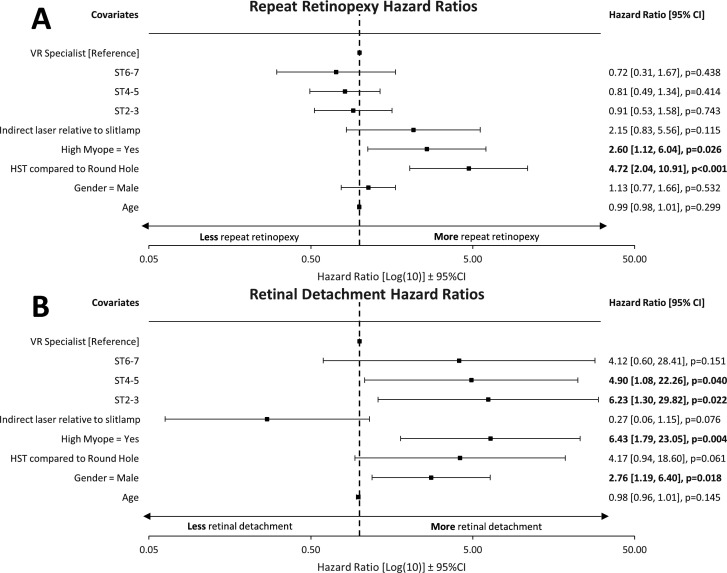
Forest plot of hazard ratios for retinal detachment and repeat retinopexy rate and training grade. Multivariable Cox regression survival model for time to repeat retinopexy and retinal detachment presented as forest plots. Significance defined as p<0.05 and highlighted in bold. (A) Repeat retinopexy: HST (relative to round holes, p<0.001) and high myopia (p=0.026) were associated with significantly higher repeat retinopexy rate. (B) Retinal detachment: male gender (p=0.018), high myopia (p=0.004), ST2-3 (p=0.022) and ST4-5 (p=0.040) (relative to VR specialists) were all associated with higher retinal detachment rate following primary retinopexy. HST, horseshoe-tear, ST, specialty trainee; VR, vitreoretinal specialist.

**Figure 2:**
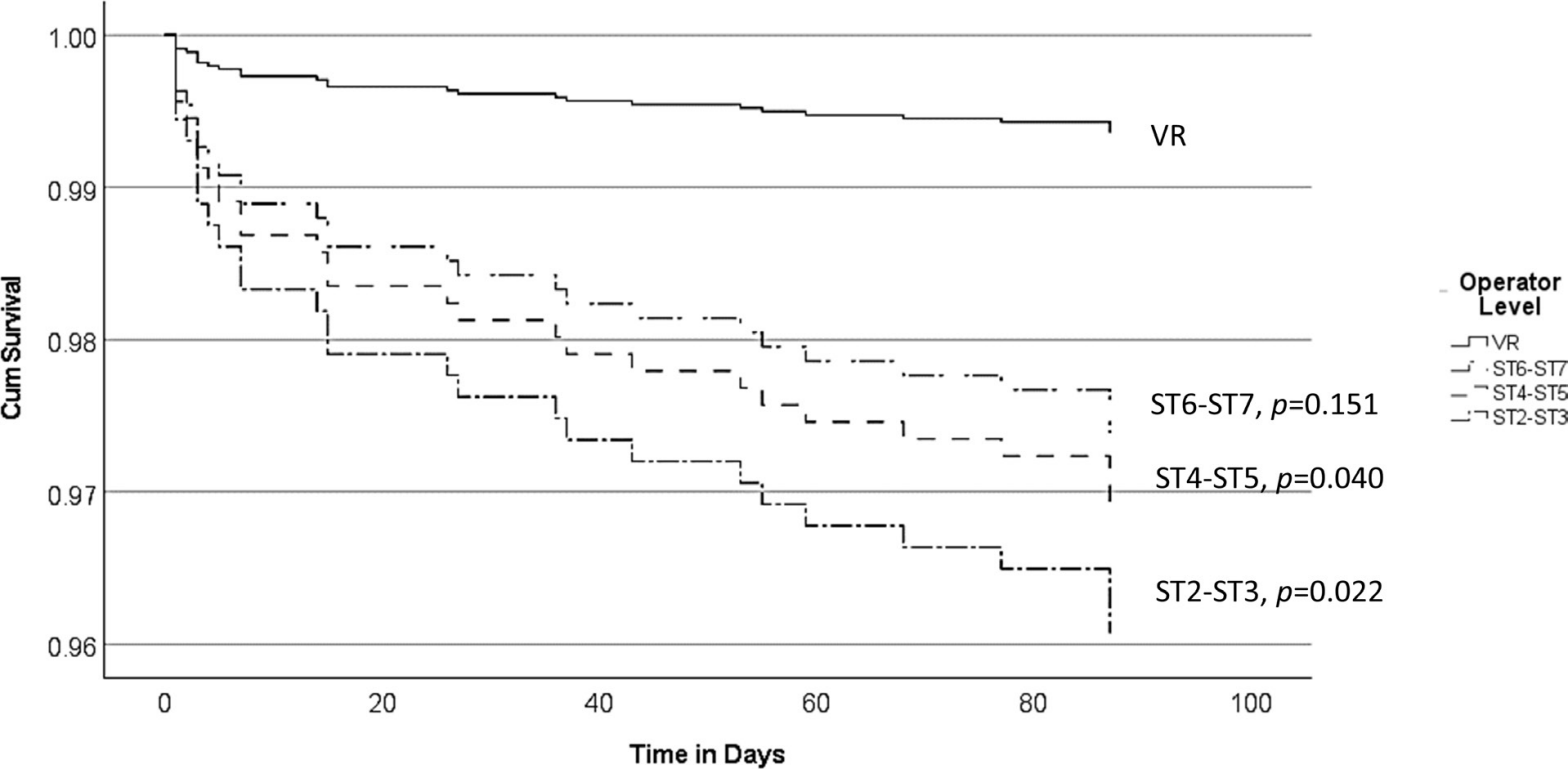
Cox proportional hazards survival plot for retinal detachment and training grade. Multivariable Cox survival regression model with dependent variable as retinal detachment within 6 months following primary retinopexy. Covariates include age, gender, high myopia, operator level, horseshoe-tear versus round hole and indirect laser versus slit lamp. ST2-3 (p=0.022) and ST4-5 (p=0.040) (relative to VR specialists) were associated with higher retinal detachment rate. ST, specialty trainee; VR, vitreoretinal specialist.

Although no significant difference was found between groups on univariate analysis, there were a high number of variable risk factors between groups. Therefore, two multivariable Cox survival regression analyses were performed on risk factors for RRD and further retinopexy following primary retinopexy to determine differences in training level as outlined in the Methods section. Our Cox regression survival analyses are found in figure 1. HST (relative to round holes, p<0.001) and high myopia (p=0.026) were associated with significantly higher repeat retinopexy rate. Male gender (0.018), high myopia (p=0.004), ST2–3 (p=0.022) and ST4–5 (p=0.040) (relative to VR specialists) were all associated with higher RRD rate following primary retinopexy. A Cox proportional hazard survival plot for RRD and training grade is found in figure 2 and a forest plot of the HRs in figure 1.

## Discussion

In our study, we explored that the influence training has on retinal detachment following primary retinopexy, the repeat retinopexy rate and the type of retinopexy performed on primary retinopexy. Through a multivariable Cox survival regression analysis, we demonstrated that increasing experience among different training grades is linked to a trend of reduced risk of developing a retinal detachment following primary retinopexy (figures 1 and 2). Our overall RRD rate of 3.3% was similar to other large case series.^3–9^ However, although we found a statistically significant reduction in RRD risk with increased experience relative to VR surgeons (figures 1 and 2), our most junior residents (ST2–3, 4.1%) still had an excellent RRD rate compared with other large case series.^3–9^ Interestingly, this finding did not apply to the number of patients requiring further retinopexy and there was no significant difference between the various training levels and VR specialists in our regression analysis. In the UK, ophthalmology residents have to complete 7 years of ophthalmology training, typically followed by 1 to 2 years of fellowship in their respective subspeciality, and this represents the longest training programme in the world. Our data are a demonstration that residents following the RCO curriculum undertaking primary retinopexy does not present a patient safety concern. The good safety profile at the start of training continuously improved over each training stage until no statistical difference was found between the most senior residents (ST6–ST7) and VR specialists in RRD rate. However, we acknowledge that our 95% CIs (figure 1) are relatively wide. Therefore, the lack of statistical significance between VR specialists and senior residents (ST6–7) should be considered acknowledging the wide 95% CIs presented in the statistical analysis. Despite this, we observed an incremental trend in reduction in detachment rate with increased level of training. Simulation training has become an increasingly important part of resident training and has been shown to improve the safety profile of junior residents performing cataract surgery.^10^ Although there are some pilot studies on the effect of laser simulation on outcomes of primary outcomes, no large data exist to date.^11^ In our region, since 2018, all new junior residents have to attend a laser simulation course. Nationally, the RCO now also mandates laser simulation training for new residents prior to using laser machines.^12^

Only a few papers investigated the effect of training on primary retinopexy in the past including a significantly lower number of patients compared with our series. In addition, none of the previous papers correlated the retinopexy outcomes to the stage of training. Unlike, in our cohort, the study by Lankry *et al*, on 307 eyes, found no significant difference in progression to RRD between residents and VR specialists.^3^ Two papers have been published in the last 20 years from our unit on trainee outcomes in retinopexy. Ghosh *et al* found a 24% repeat retinopexy rate and a RRD rate of 8% (treated by scleral buckle) following primary retinopexy by resident ophthalmologists.^4^ Petrou *et al*, 10 years following Ghosh *et al*, found that 40% of eyes lasered by residents in our unit subsequently required further laser, with no patients developing a RRD.^5^ In our cohort, the repeat retinopexy rate in the residents’ group was lower at 19.8%. While the prior studies in our unit only considered 100 consecutive cases presenting to the eye emergency department (EED), our case series is 10 times larger and more representative of subsequent retinopexy following primary treatment across the entire department, as we included the cases that presented to all clinics. Additionally, laser simulation may have led to improved performance of junior residents in our region. Levin *et al* reported a 15% retreatment rate and a 1.2% RRD rate following retinopexy.^13^ However, only 43.4% of their patients were symptomatic, which makes their cohort not comparable to ours, which includes mostly cases presenting acutely to the EED.

We also demonstrated a difference in mode of retinopexy performed between VR specialists and residents, with significantly less indirect retinopexy being performed by the latter group as already showed by Ghosh et al. almost 20 years ago.^4^ This is not surprising as indirect laser retinopexy is a complex technique which requires the correct application of various skills simultaneously, including indirect ophthalmoscopy and scleral indentation. Although indirect retinopexy is not part of the ST curriculum, our study showed that further scope remains within our unit for supervised training in laser indirect ophthalmoscopy as some pathologies cannot adequately be treated with slit-lamp retinopexy alone.^14^

### Study limitations and strengths

Our study is retrospective in nature and so we had no standardised treatment protocol to follow. We used RRD surgery as a rate of failed primary retinopexy at 6 months. Although patients may have had their surgery at another eye unit, patients were excluded by postcode outside of our catchment area to minimise this probability. The presence of subretinal fluid (SRF) was not consistently reported and was not included as a risk factor in our analysis. However, tears with extensive SRF, (localised retinal detachment) were all subcategorised and presented in table 1. All our groups had above 200 retinopexies performed apart from our ST6 to ST7 group at n=72, meaning this group is relatively underrepresented in our cohort. This is due to the different clinical duties undertaken by more senior residents in our unit. Our study also has several strengths. We collated the largest case series on this topic to date which enabled us to assess the primary retinopexy outcomes by training level at one of the largest teaching hospitals in the UK. Additionally, a multivariable analysis helped to reduce confounders of multiple investigators with various VR experience, multiple mode of delivery of treatment, and variations in clinical presentations.

## Conclusions

Overall, we discovered a decreasing RRD rate following primary retinopexy with the increase in training grade and experience. Although junior residents had a higher RRD rate than VR specialists, this was still favourable compared with other large case series. We demonstrate an incremental reduction in detachment rate with increased training. Although VR specialists on multivariable analysis maintained the lowest detachment rate across the cohort, the most senior residents showed no statistical difference in RRD rate compared with VR specialists, relative to their more junior colleagues, demonstrating the effectiveness of the RCO training curriculum.

## Data Availability

Data are available upon reasonable request.
